# Permeable Nanomontmorillonite and Fibre Reinforced Cementitious Binders

**DOI:** 10.3390/ma12193245

**Published:** 2019-10-04

**Authors:** Styliani Papatzani, Sotirios Grammatikos, Kevin Paine

**Affiliations:** 1 Department of Mathematics and Engineering Sciences, Hellenic Army Academy, Evelpidon Avenue, 166 72 Attika, Greece; 2 Hellenic Ministry of Culture, Directorate for the Restoration of Byzantine & Post-Byzantine Monuments, Tzireon 8-10, 11742 Athens, Greece; 3 Group of Sustainable Composites, Department of Manufacturing and Civil Engineering, Norwegian University of Science and Technology, 2815 Gjøvik, Norway; sotirios.grammatikos@ntnu.no; 4 BRE Centre for Innovative Construction Materials, University of Bath, BA2 7AY Bath, UK; k.paine@bath.ac.uk

**Keywords:** Portland limestone fibre-cement binders, nanomontmorillonite, PVA fibres, flexural strength, TGA/dTG, XRD, MIP, water impermeability tests

## Abstract

Clinker reduction in cementitious binders is of paramount importance today, and nanotechnology has extended permissible limits. In the present study, a reference binder consisting of 60% Portland cement, 20% limestone, 20% fly ash, 3% polyvinyl alcohol (PVA) fibres and 2% superplasticizer is optimized with three different types of nano-montmorillonite (nMt) dispersions; two organomodified ones and an inorganic one at different proportions (0.5% to 4%). Flexural strength, measured on day 7, 28, 56 and 90, was improved after day 28 with the addition of inorganic nMt. Thermal gravimetric analyses carried out on day 7, 28, 56 and 90 coupled with x-ray diffraction (at day 28) showed a distinctively enhanced pozzolanic reaction. Backscattered electron imaging confirmed changes in the microstructure. Late age relative density measurements of the nMt cementitious nanocomposites showed higher values than these of the reference paste, which can be attributed to better particle packing. Mercury intrusion porosimetry measurements give support to the optimal nMt dosage, being 1% by total mass of binder and water impermeability tests (modified with BS EN 492:2012) suggest that inorganic nMt can be a viable option material where permeability constitutes a prerequisite. Suggestions for further activation of the nMt-fibre reinforced cementitious nanocomposites were also made.

## 1. Introduction

Nanotechnology continues to reshape the world of construction materials, with the extensive selection of experimental techniques for monitoring the matter at the nanolevel and with the wide variety of nanoparticles available [1,2,3]. In cement research, nanosilica [4,5,6], nanomontmorillonite (nMt) [3,4,7,8] or carbon-based nanoparticles [9,10] are amongst the most effective nanoparticles implemented in innovative formulations for various tailor-made applications, functioning as fillers, nanoreinforcement or nano-additives. At the same time, clinker is the main constituent of all Portland cement blends, but its production through the clinkering process accounts for approximately 8% of global manmade CO_2_ emissions. Reducing clinker and introducing supplementary cementitious materials (SCM) has, therefore, become imperative in the quest for greener construction materials and applications. On the other hand, some of the most broadly researched SCMs, such as fly ash and blast furnace slag, are being depleted in a number of countries [11]. The case of limestone is of particular interest due to the low cost and the near zero emissions, as well as that of activated clays [12]. It should be noted that according to BS EN 197-1:2011 [13], for CEMII/A-L cements, the allowable amount of Portland cement clinker (PC) must fall within 80–95% by mass of binder and that of limestone (LS) must fall within 6–20%. Similarly, according to BS EN 197-1:2011, for the Portland-composite cements, i.e. for formulations that also contain fly ash and limestone at a total maximum of 35%, clinker should be at least 65% by total mass of binder [13]. It has been postulated that if clinker is reduced and SCMs are increased a number of issues may arise with the performed mixes such as reduced compressive or flexural strengths, prolonged setting times, voids in the microstructure [5]. 

At the same time, it is commonly accepted that the dimensions of the main hydration product, calcium-silica-hydrate (C–S–H), fall in the nanoscale (below 10^−9^ m) [2] and nanosilica or nMt is of similar dimensions [14], therefore potentially suitable for use in cementitious binders. The design idea elaborated upon in a number of studies is then expressed with the following questions: “can we leverage the adverse effects of very low PC and very high SCM content with the use of nanotechnology, which nanoparticles should be used, at which concentrations and/or potential combinations and which would be the suitable applications?”.

**Figure 1:**
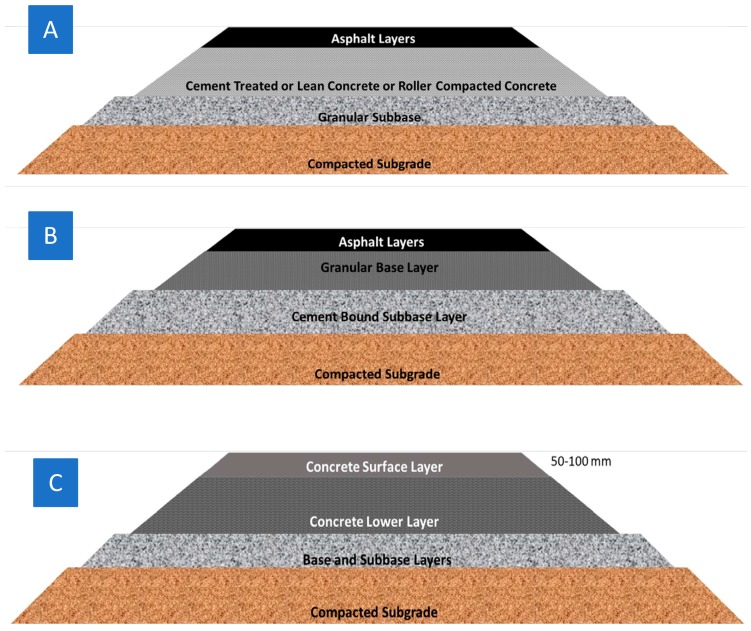
Cross sections of (**A**) a semi- rigid pavement, (**B**) an inverted pavement and (**C**) composite two-lift pavement [21].

In addition, the development of pervious concrete pavements has been proposed as a solution to stormwater management [15] and urban flooding [16], possibly achievable with the use of nanoclays [17]. Permeable pavements include “porous asphalt pavements, pervious concrete pavements, pervious cast concrete pavement, permeable interlocking concrete pavements, and various types of permeable gravel pavements” [18]. Recent research on the life cycle impact of permeability of permeable pavements stresses the need to address traffic delay and congestion, damage to infrastructure and even loss of lives as a result of urban flooding. Moreover, it explains how sinking of the city ground can be eliminated by the infiltration of stormwater through permeable pavements, concluding that the prime advantage of dense-graded semi-rigid asphalt pavements have over permeable asphalt pavements is the better mechanical performance [16]. In the United States, the Environmental Protection Agency (EPA) has established regulations following which approximately 75% of urban surface area is covered by impermeable pavement [19]. The runoff is managed either by the construction of best management practices (BMPs) or of sustainable urban developments (SUDs) [18]. The latter include fully permeable pavements for streets and highway shoulders which reduce storm-water runoff. Hence, water pollution is also reduced, ground water is recharged and wastewater/rainwater is reused [20]. Pervious concrete is ideal for structures that are not constantly heavily loaded such as trails, sidewalks, driveways and parking lots. Apart from concrete pavements also known as rigid or semi-rigid pavements (Figure 1A), which combine both asphalt and concrete (semi-rigid) layers, cement can be used as a stabilizing agent for base and subbase layers for inverted pavements (Figure 1B) [21]. Lastly, an innovative two-lift pavement has been presented comprising a thin concrete overly and a thick concrete base layer. (Figure 1C) [21]. 

There is extensive room for improvement of the cementitious matrix of the concrete formulations suitable for pavements in light of the necessity for greener solutions. Nanoclays, and particularly nMt, possess the additional property of removing organic pollutants [22] and therefore constitutes one of the best candidate nanoparticles for implementation in pervious formulations. Recent research has proven the pozzolanic properties of nMt [23] and the potential for higher flexural strength achieved by addition of 1% nMt by total mass of solids in ternary blends [24]. In a paper published by the authors, it has been demonstrated that the pore structure of ternary nanocomposites can be enhanced by the addition of nMt [8]. In addition, given that the nMt is comprised of either exfoliated or/and intercalated platelets, it has been found that their orientation may influence the way cracks are formed within the mass of the hydrating cementitious matrix [25]. Furthermore, during stirring of the mix, nMt particles may further exfoliate and orient in a specific direction [26]. This in turn, may favour improvements in flexural, rather than compressive strength. Lastly, the damping properties of the MMT’s have been confirmed [27] by dynamic mechanical analysis (DMA) opening horizons on the use of these materials in applications where damping is required. 

**Figure 2:**
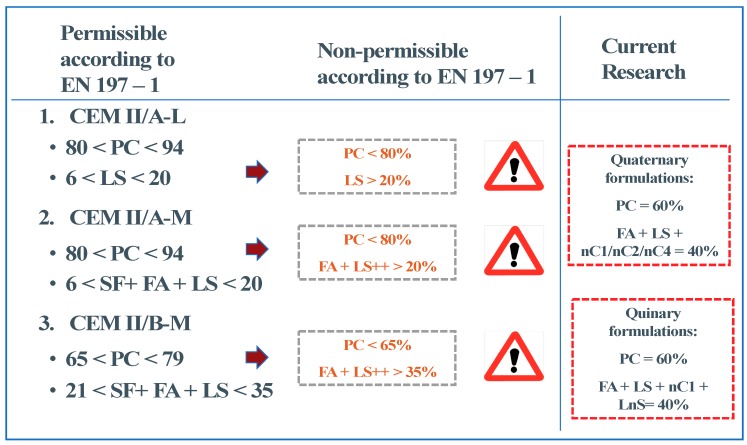
Eurocode limits of clinker substitution and supplementary cementitious materials (SCM) addition and target of current research.

In this paper, the reduction of Portland cement blinker by up to 20% by total mass of solids compared to the allowable limits set by the Eurocodes (Figure 2) has been extended with the development of durable pervious nMt quaternary and quinary fibre binders. In this study three different types of nMt dispersions have been investigated; an organically modified (OMMT) one dispersed with non-ionic fatty alcohol surfactant, an organically modified one dispersed with an anionic alkyl aryl sulfonate surfactant and an inorganic one [25]. Although previous research has found that the 1% replacement tends to be optimal [3,24], the current research investigates the effect over a range of nMt additions in order to investigate further activation potentials in fibre reinforced nanocomposites. The research carried out is the first presented on the enhancement of quaternary and quinary fibre reinforced binders themselves. In fact, very few papers have been published to date on the use of nMt as supplementary cementitious materials all of which on binary binders, except for one on ternary combinations [28]. This way, the effect of a number of constituents in the nanocomposites was investigated and the potential of achieving additional permeability by adding nanoparticles in fibre cements was explored well before aggregates are added. 

## 2. Materials and Methods

### 2.1. Materials

The following materials were used:

- Portland limestone cement CEMII/A-L42.5, with a limestone content of 14%, conforming to EN 197-1. The supplier gave the following clinker composition: 70% C_3_S, 4% C_2_S, 9% C_3_A, 12% C_4_AF.
- Limestone (LS) (additional), conforming to EN 197-1.
- Fly ash (FA), conforming to EN 450. The oxide composition provided by the material data sheet was: 53.5% SiO_2_, 34.3% Al_2_O_3_, 3,6% Fe_2_O_3_, 4.4% CaO.
- Organomodified nMt, nC1 dispersed in water with the help of a non-ionic fatty alcohol and 1% by mass defoaming agent, containing about 15% by mass of nMt particles.
- Organomodified nMt, nC2 dispersed in water with the help of an alkyl aryl sulfonate surfactant, containing about 15% by mass of nMt particles.
- Inorganic nanomontmorillonite, nC3, in an aqueous dispersion containing about 15% by mass of nMt particles.
- Commercially available colloidal amorphous nS 15% by mass of nanoparticles in an aqueous suspension (LnS)
- Polyvinyl alcohol (PVA) fibers, kuralon H-1, 4 mm added at 2% by weight in quaternary pastes and at 4% by weight in quinary pastes.
- Superplasticizer (SP) viscocrete 20HE added at 2% by weight.

The organomodified and inorganic nMt dispersions have been characterized elsewhere [7,8,23,25], however the main characteristics they encompass, relating to the study presented herein, are the following:

- In nC1 the platelets were possibly re-agglomerating in the cement paste. This re-agglomeration leads to increase in porosity and reduction in density of nC1 added formulations due to the void creation. Such results were expected to aggravate with the increase of the nC1 percentage addition.
- In dispersion nC2, the anionic surfactant mostly kept the platelets partially exfoliated, better dispersed in water and did not allow the micro-cracks to propagate under loading conditions. Some areas of re-agglomeration were identified.
- Dispersion nC3 was fully exfoliated allowing for centers of crystallization to form and bridge or arrest micro-cracks from forming.

### 2.2. Mix Design

For all specimens produced the water to solids ratio (w/s) was kept constant and equal to 0.3. It is worth noting that the PC content was also kept constant and the content of nMt solids was deducted from the LS content. This was done in order to keep the Ca(OH)_2_ production, during PC hydration comparable in all pastes, so as to detect additional pozzolanic reactivity of the nanoparticles or hindrance of pozzolanic reactions in these composite cement formulations. Continuing from previous research on ternary, non-pozzolanic formulations in terms of the reference paste [28], this research program aimed to investigate the combined effect of fly ash, limestone, nMt and fibres in higher order formulations. In the present paper a quaternary formulation comprising a pozzolanic reference paste containing 60% PC, 20% LS, 20% FA, 3% PVA fibres and 2% superplasticizer, denoted as F.PC60LS20FA20PVA3SP2 (F is for flexure) is enhanced with nMt and a quinary formulation comprising a pozzolanic reference paste containing 60% PC, 20% LS, 20% FA, 4% PVA fibres and 2% superplasticizer, denoted as F.PC60LS20FA20PVA4SP2 is enhanced in a step by step manner initially by adding nMt and consequently by adding nMt and LnS. 

#### 2.2.1. nMt-fibre Reinforced Quaternary Cementitious Nanocomposites

The formula for the nMt-fibre reinforced quaternary matrix was:

PC (60) + LS (20 − x) + FA (20) + PVA (3) + SP (2) + xnMt(1)

where x = % of nMt solids.

The different concentrations of the nMt dispersions are shown in Table 1. The full series with all four different nMt concentrations were created only for nC1 and nC2. For the nC3 only the 1% concentration was implemented. The amount of 2% superplasticizer was tested in previous research [28] and was found to be ideal in ternary formulations which also contained fibres and nMt, therefore the quaternary ones also started off with 2% superplasticizer.

#### 2.2.2. nMt and LnS-fibre Reinforced Quinary Cementitious Nanocomposites

The work conducted for the quaternary binders was then extended to quinary binders, having as a reference paste F.PC60LS20FA20PVA4SP2, enhanced by the addition of two different nanoparticles; nC1 and nanosilica (LnS) as shown in Table 2. These series were only tested in flexure and no further characterization was carried out. It served as an indication for directing future work suggestions. Further aims of this limited study included the assessment of the possibility of achieving higher flexural strengths by adding greater amounts of PVA fibres, without compromising the miscibility of the pastes and to assess the combined effect of the addition of nanosilica and nanomontmorillonite particles.

The formula for the nMt-fibre reinforced quinary matrix was:

PC (60) + LS (20 − x − y) + FA (20) + PVA (4) + SP (2) + xnMt + yLnS(2)

where x = % of nMt solids, y = % of LnS solids.

### 2.3. Sample Preparation

#### 2.3.1. Production of Specimens

**Figure 3:**
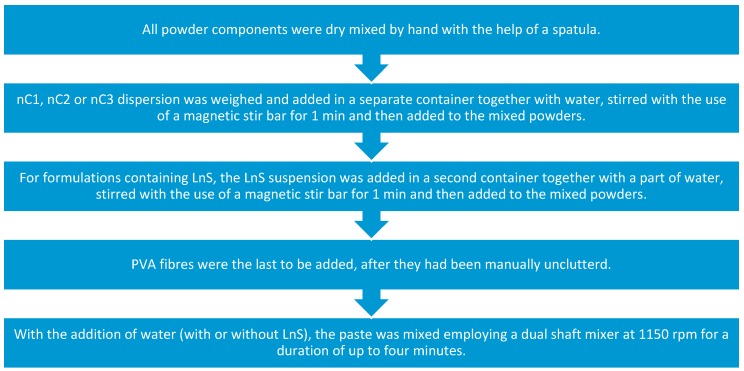
Flow chart of the mixing procedure.

The mixing procedure was standardized as in the case of research on nanosilica particles presented by the authors [6] and is summarized in Figure 3.

**Figure 4:**
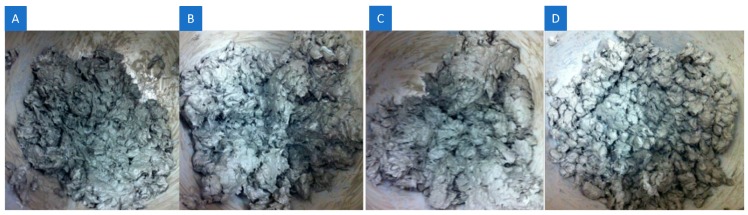
(**A**) 0%, (**B**) 0.5%, (**C**) 1% and (**D**) 4% by mass addition of nC2 on F.PC60LS20FA20PVA3SP2.

As the amount of nMt increased, the pastes showed signs of re-agglomeration and increasing compaction difficulty (Figure 4).

**Figure 5:**
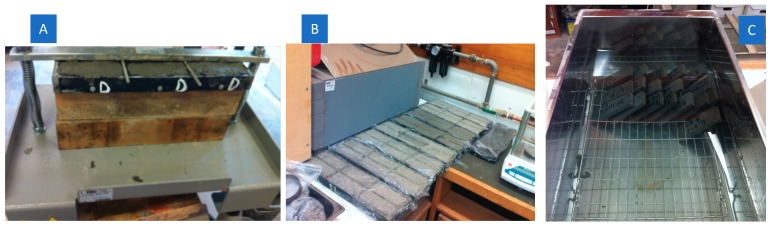
(**A**) Compacting pastes at shaking table, (**B**) initial 24-h air curing and (**C**) water curing thereafter.

Pastes were cast in prismatic moulds, producing slabs of the following dimensions: 10 mm depth, 40 mm breadth and 120 mm length and compacted at a shaking table (Figure 5A). They were subjected to a 24-h air-curing (Figure 5B) and subsequently demoulded and cured in distilled water thereafter at 20 ± 2 °C (Figure 5C) until the day of mechanical testing.

#### 2.3.2. Sample Preparation for Characterization.

From the abovementioned production process samples were also produced for characterization. After the desired water curing was achieved, hydration was stopped using two different methodologies: oven drying and solvent exchange as described by Calabria-Holley et al. [29]. Oven drying was preferred for the thermogravimetric/differential thermogravimetry analysis (TGA)/dTG analysis and solvent exchange for the mercury intrusion porosimetry (MIP) testing. Isopropanol was selected as the most appropriate solvent according to literature [30,31].

### 2.4. Testing Programme

Flexural strength tests were carried out in accordance with BS EN 12467. Mean strength values of at least three tests per formulation were calculated. All samples were tested at a loading speed of 0.5 MPa/s on a Dartec 100 kN servo hydraulic testing machine (Dartec, Stourbridge, U.K.).

Thermogravimetric analysis (TGA) was carried out using Setaram TGA92 (Setaram, Caluire, France). Each powder sample was placed in an alumina crucible and heated at a rate of 10 °C/min from 20 °C to 1000 °C in nitrogen atmosphere. Three areas were monitored with the TGA/dTG; (i) the area between 100 °C and 180 °C, which corresponds to the dehydration of C–S–H, ettringite and monosulfate, (ii) the area between 400 °C and 500 °C, which corresponds to the dehydration of Ca(OH)_2_ and (iii) the area 600 °C and 800 °C, which corresponds to the decomposition of CaCO_3_.

A D8 ADVANCE X-ray diffractometer (Bruker, Coventry, U.K.) with CuKα radiation was used for the X-Ray Diffraction (XRD) analyses. Spectra were obtained in the range 4° < 2θ < 60°. Bragg’s law (nλ = 2dsinθ) was used for determining peaks and d-spacing with the help of EVA software.

A set of scanning electron microscopy (SEM) images was taken for the reference paste and for the 1% or 4% addition of nC1 and nC2, at day 28. Samples were imaged using a Jeol 6480 LV SEM, Jeol, Herts, U.K., UK. Backscattering was used to capture images of the as received and uncoated, samples.

BS EN  12390-7:2009 (BSI, 2009), was used as a reference for the late age relative density measurements. The modifications in the code prescribed methodology, is mentioned in other publications [4]. Practically, water displacement was used for the derivation of the volume:

V = (m_a_ − m_w_)/ρ_w_ (3)

where, m_a_ = the mass of the specimen in air, in kg, m_w_ = the apparent mass of the immersed specimen in water, in kg, ρ_w_ is the density of water, at 20 °C, taken as 998 kg/m³.

Moreover,

D = m_a_/V (4)

where, D is the relative density in kg/m^3^.

Autopore III—Model unit 9420 supplied by Micromeritics, (Hexton, Herts, U.K.) was utilized for the mercury intrusion porosimetry (MIP) tests. Solids of the arrested hydration pastes were placed in 3 mL stems.

The modified water impermeability test (BS EN 492:2012) was performed on slabs 120 mm × 40 mm and 10 mm thickness. According to the methodology proposed, a 250 mm long transparent tube with an internal bore of 29 mm diameter, was attached to the specimens using a sealant. A control water column was placed next to the specimens with the attached tube to ascertain that no water evaporation would take place in the laboratory testing environment [28].

## 3. Results and Discussion

### 3.1. Flexural Strength of nMt and Fibre Reinforced Cementitious Nanocomposites

#### 3.1.1. nMt-fibre Reinforced Quaternary Cementitious Nanocomposites

**Figure 6:**
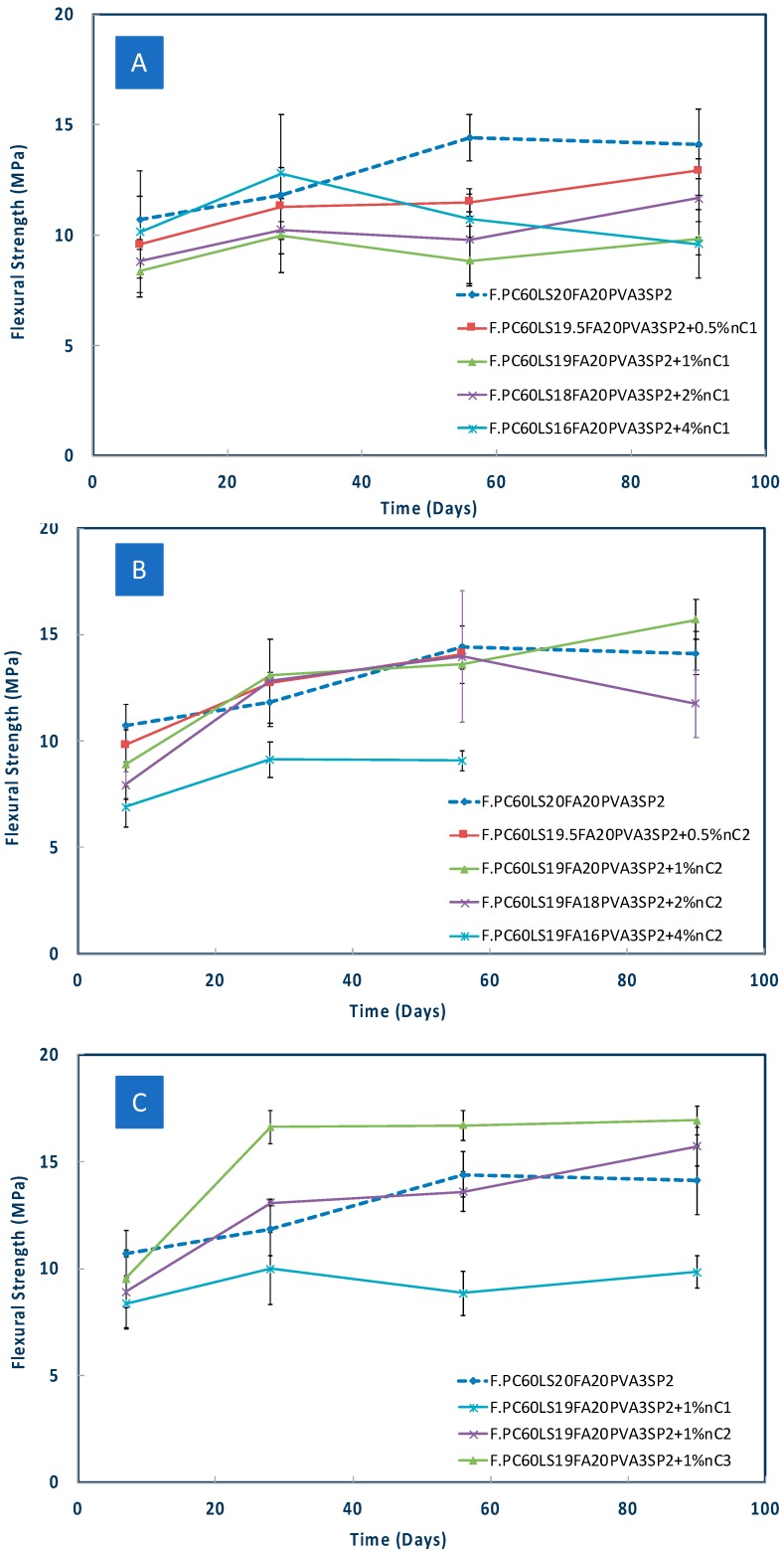
Flexural strength of (**A**) nC1, (**B**) nC2 fibre reinforced cementitious nanocomposites based on F.PC60LS20FA20PVA3SP2 (**C**) comparison of flexural strength of 1% nC1, nC2 and nC3 fibre reinforced cementitious nanocomposites.

It can be seen in Figure 6A that nC1 was unable to deliver higher strengths compared to the reference paste at any dosage. 

As shown in Figure 6B, for nC2, only the 1% by mass addition offered optimal results. The lowest, 0.5% by mass, nMt dosage did not offer any flexural strength improvement, whereas the highest, 4%, dosage, proved to be the most detrimental of all. 

In a previous extended study carried out by the authors, on ternary formulations containing 60% PC and 40LS as a reference binder, nC1 and nC2 offered limited compressive strength improvements and this was partly attributed to technical difficulties with the mixing of the pastes [32], as the nMt dispersions increased the viscosity of the pastes, even though the binders did not contain fibres. The following were, hence, advised: mixing for longer time; better dispersion of the nanoparticles within the paste; better compaction, therefore, greater homogeneousness of the paste; and better particle interaction, limiting air voids. In addition to this, the use of superplasticizers was also offered as an alternative in order not to increase the water content of the formulation. In the present research, superplasticizer was in fact used, however nC1 again did not offer strength improvements and the effect of the addition of nC2 was marginal for the 1% addition. Only nC3, which has been reported in the past as highly reactive [8,24,32] offered significant strength improvements, as shown in Figure 6C.

It is also worth mentioning that the standard deviation for the reference paste ranged from 1 to 1.2 MPa, however it was similar for the nC1 and nC2 modified samples, reaching approximately 1.2 MPa, whereas for nC3 it was much lower again and equal to about 0.8 MPa.

The limited performance of the organomodified nanomontmorillonites has been connected to the production method of the material itself [25] and the inherent incompatibility with the cement paste, which is inorganic. It has been postulated that the electrical charge within the hydrating cement paste alters the charge of the organomodified montmorillonite when it is added to the paste, which in turn re-agglomerates and creates some zones of weakness, which can also increase the porosity [8]. The inorganic nMt, instead, has been reported to provide compressive strength enhancement [32] and flexural strength enhancement in ternary nanocomposites [28].

#### 3.1.2. nMt and LnS-fibre Reinforced Quinary Cementitious Nanocomposites

**Figure 7:**
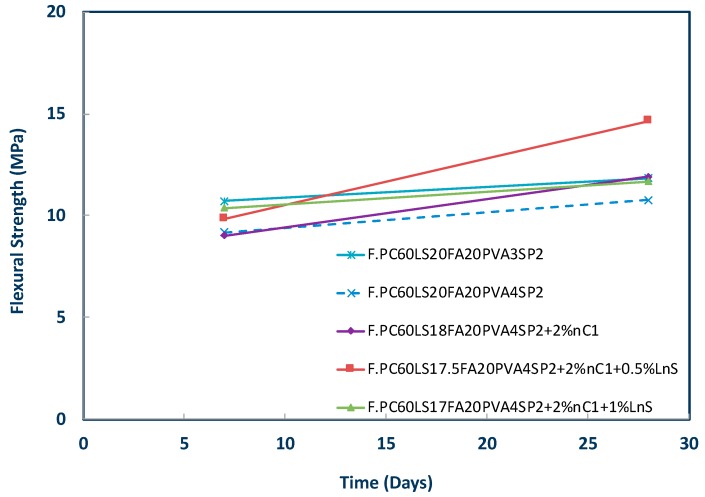
Flexural strength of nC1 and nanosilica (LnS) and fibre reinforced cementitious nanocomposites based on F.PC60LS20FA20PVA4SP2.

These series constitute a step by step process of activation of the less favourable nMt dispersion, nC1. As a first step, the PVA fibre content was increased from 3% to 4% in an effort to enhance the flexural strength. Then, in a subsequent mix 2% of nC1 was added. Next, 0.5% LnS was also added to the 2% nC1 and 4% PVA matrix and as a last step 1.0 % LnS was added to the 2% nC1 and 4% PVA matrix. Nanosilica and in specific aqueous nanosilica rather than polycarboxylate nanosilica was found to offer significant strength improvements in cementitious nanocomposites as reported in previous research [3,4,28,29]. For this, nanosilica (LnS) was added at two different dosages. Once again, it can be observed that the 0.5% by mass LnS addition is more effective in terms of strength gain than the higher LnS addition, that is, 1% by mass of binder (Figure 7). It is acknowledged that F.PC60LS20FA20PVA4SP2 is by no means a complete series if no later ages are studied and also a comparison between the effect of the difference nMt dispersions. It can also be seen in Figure 6 that the higher PVA content caused a reduction in strength possibly due to the mixing and compaction difficulty. However, the addition of nC1 and LnS offered a 50% strength increase by day 28. This can be attributed to the increased pozzolanic activity that LnS is offering.

The purpose of these series was to set the ground for suggestions for “further research”, presented in paragraph 4 of this paper. For this reason, no further investigations of these pastes have taken place at this stage. The results, however, clearly show the activation of even the less favourable nMt dispersion, nC1, by the addition of LnS, which is in full agreement with research on binary combination of nanomaterials in concrete specimens [33].

### 3.2. Thermogravimetric and Crystallographic Analyses of nMt and Fibre Reinforced Cementitious Nanocomposites Based on F.PC60LS20FA20PVA3SP2

**Figure 8:**
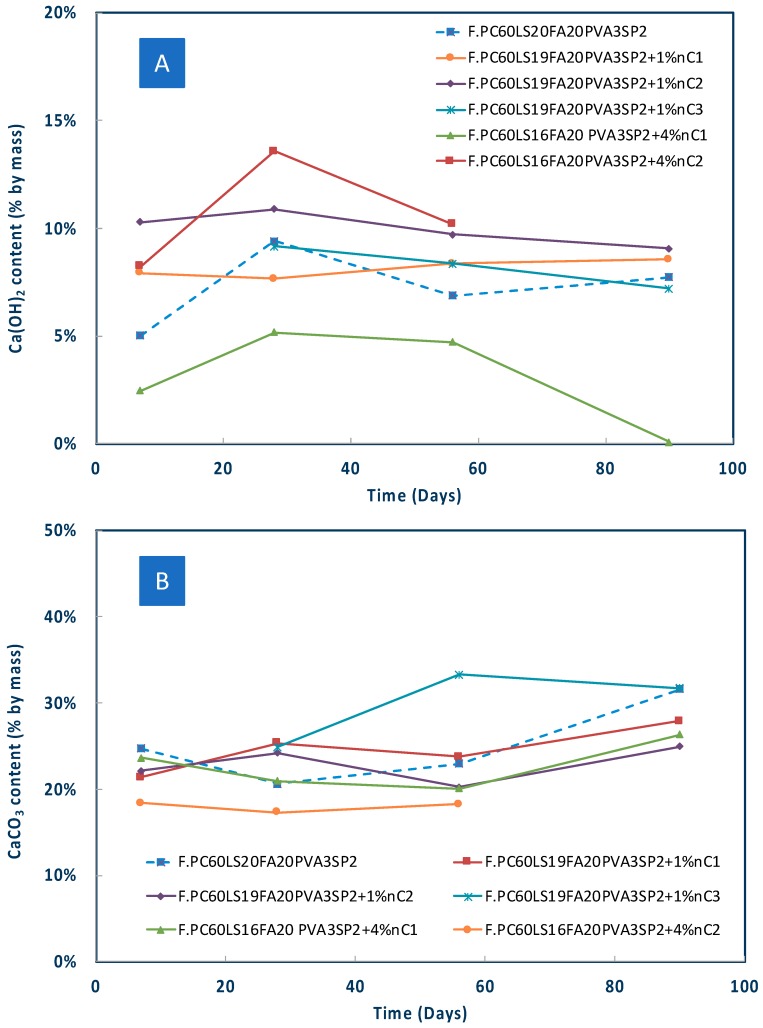
Effect of nMt content and type on (**A**) Ca(OH)_2_ and (**B**) CaCO_3_ content of nMt and fibre reinforced cementitious nanocomposites based on F.PC60LS20FA20PVA3SP2.

**Figure 9:**
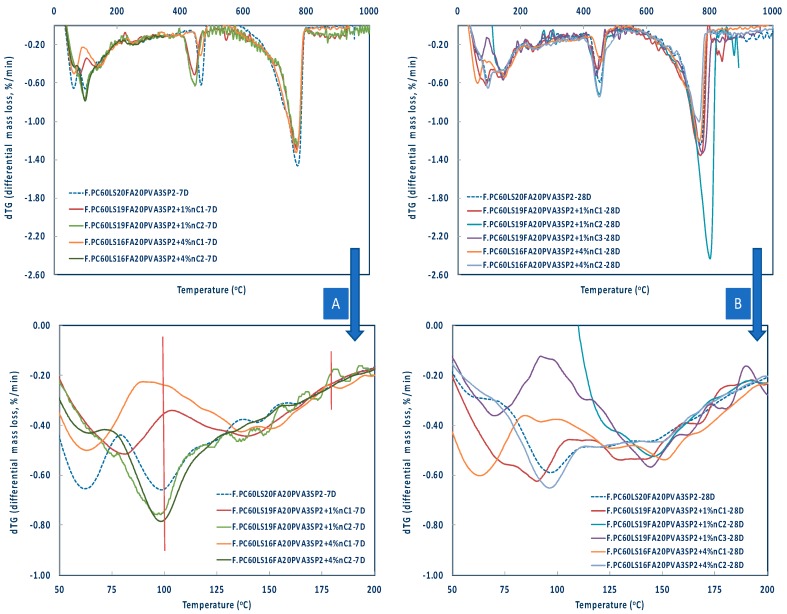
Differential mass loss between 100–200 °C of nMt and fibre reinforced cementitious nanocomposites at (**A**) Day 7 and (**B**) Day 28.

**Figure 10:**
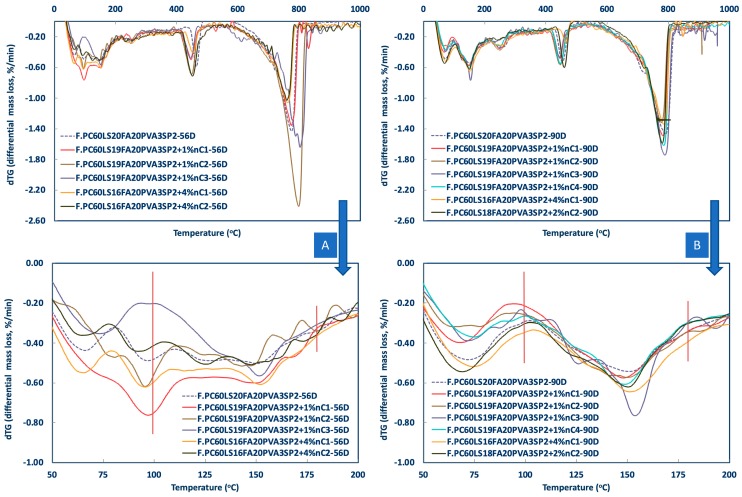
Differential mass loss between 100–200 °C of nMt and fibre reinforced cementitious nanocomposites at (**A**) Day 56 and (**B**) Day 90.

The results of the thermogravimetric analysis are shown in Figure 8, Figure 9 and Figure 10. The pozzolanic performance was evaluated by TG analyses between approximately 100–180 °C, where the decomposition of C–S–H takes place [5] and at different ages, different nMt types, and percentages of nMt content.

As indicatively shown, in the first 28 days nC1 and nC3 had a more pronounced effect (Figure 9), whereas by day 90 (Figure 10) nC3 produced the greatest quantities of ettringite and C–S–H, followed by nC2 and nC1. The latter two, performed better at higher nMt concentrations (4%) rather than 1%.

There is a possibility of carbonation taking place by day 28. This can be attributed to the high early-days porosity of the of nMt and fibre reinforced cementitious matrix, which “facilitates the penetration of CO_2_ within the composite” [34]. However, as hydration reactions continue beyond day 28, the matrix densifies and carbonation is not present any more.

**Figure 11:**
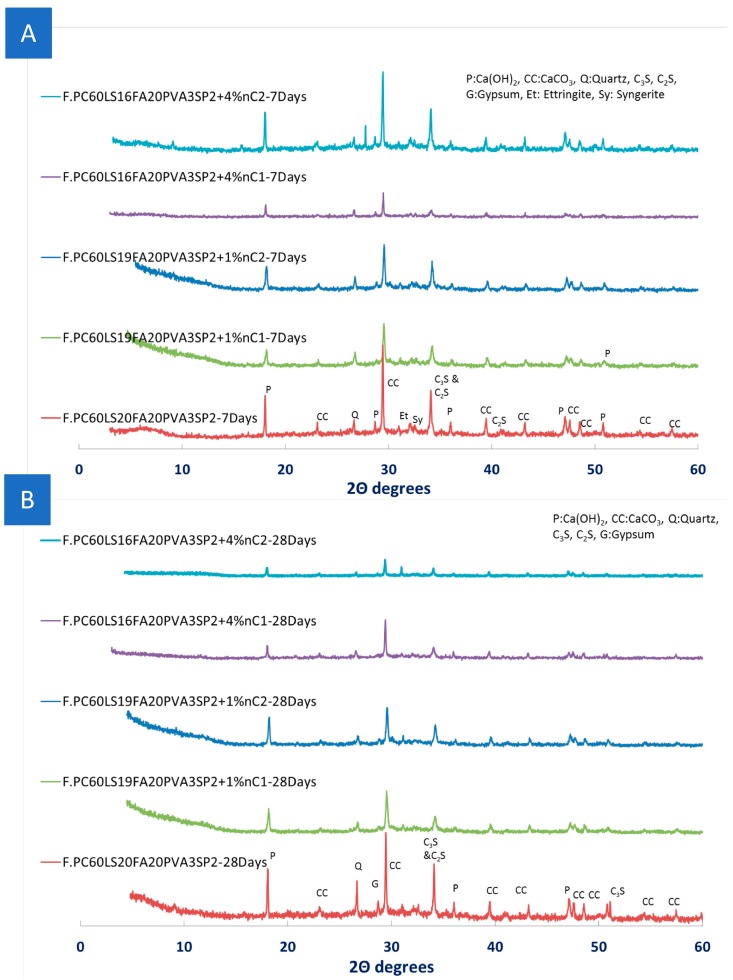
XRD pattern of nMt and fibre reinforced cementitious nanocomposites at (**A**) Day 7 and (**B**) at Day 28—Effect of nC content and type.

Since the consumption of Ca(OH)_2_ could not be confirmed by TGA, XRD measurements were carried out for two different concentrations 1% and 4% of the organomodified nMt’s. As early as seven days, the consumption of Ca(OH)_2_ was confirmed (Figure 11A) by both types of nMt, even at the 4%. The nC1 or nC2 and fibre reinforced nanocomposites exhibited much lower intensity Ca(OH)_2_ diffraction peaks. Additionally, it can be seen that CaCO_3_ was consumed. As the time advanced, the consumption of Ca(OH)_2_ towards production of additional was more distinct (Figure 11B).

**Figure 12:**
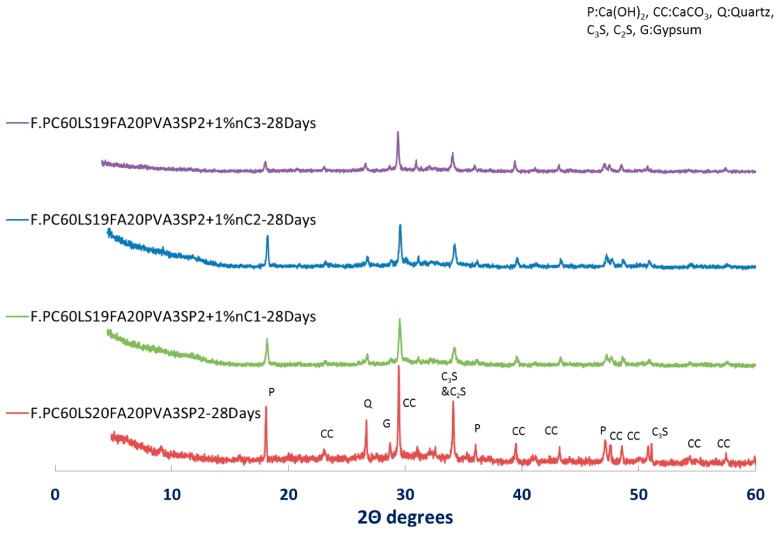
XRD pattern of 1% nMt and fibre reinforced cementitious nanocomposites at day 28—Effect of nMt type.

Lastly, as clearly shown in Figure 12, for the same amount of nMt in the same reference paste, nC3 exhibited the greatest Ca(OH)_2_ consumption. Therefore, this reference fibre cement paste seems to be exhibiting the best performance in terms of pozzolanic reactivity of the nMt. This conclusion is in agreement with research on binary formulations [26].

### 3.3. Microstructural Characterisation of nMt and Fibre Reinforced Cementitious Nanocomposites Based on F.PC60LS20FA20PVA3SP2

**Figure 13:**
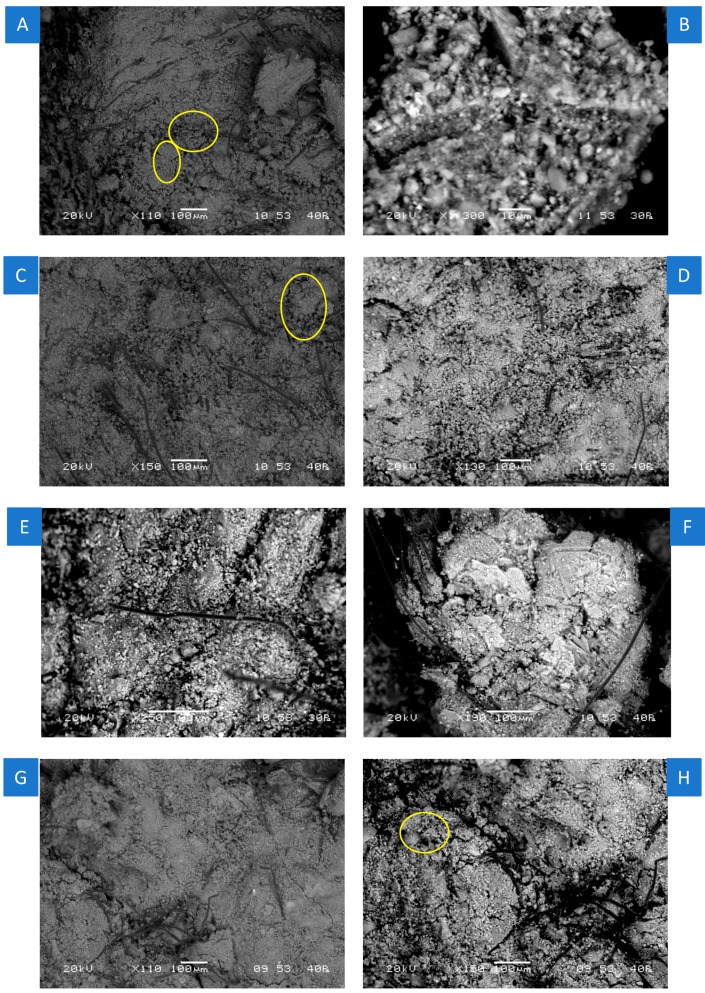
Backscattered scanning electron microscopy imaging (BSE) micrograph of (**A**) F.PC60LS20FA20PVA3SP2 at 110× magnification (**B**) F.PC60LS19FA20PVA3SP2 + 1% nC1 at 1300× magnification, (**C**) F.PC60LS19FA20PVA3SP2 + 1% nC2 at 150× magnification, (**D**) F.PC60LS16FA20PVA3SP2 + 4% nC1 at 130× magnification, (**E**) of F.PC60LS16FA20PVA3SP2 + 4% nC1 at 250× magnification, (**F**) of F.PC60LS16FA20PVA3SP2 + 4% nC1 at 190× magnification, (**G**) F.PC60LS16FA20PVA3SP2 + 4% nC2 at 110× magnification and (**H**) F.PC60LS16FA20PVA3SP2 + 4% nC2 at 150× magnification.

The investigation in the morphology of the nMt modified fibre cement pastes based on F.PC60LS20FA20PVA3SP2 at day 28 is portrayed in Figure 13, with the use of backscattered scanning electron microscopy imaging (BSE). For this reason, the selected magnifications were primarily low. The reference paste (Figure 13A) exhibited a grainy surface and clustering of materials probably due to the mixing, which left localised voids. The reference paste looked very dense, overall and the fibres seemed to be dispersed in all directions, binding the paste. Some differences can be observed between the two nMt types, nC1 and nC2 and the two nMt dosages 1% and 4%. nC1. It can be claimed that nC2 and lower nMt additions delivered visually denser pastes. A number of reacted FA particles were identified and marked in a circle. It can be argued that nC1 made the formulation more brittle, a fact more evident at higher nC1 dosages (Figure 13E,F), in which microsized clusters of materials are predominant, together with microcracks.

### 3.4. Late Age Relative Density of nMt and Fibre Reinforced Cementitious Nanocomposites Based on F.PC60LS20FA20PVA3SP2

**Figure 14:**
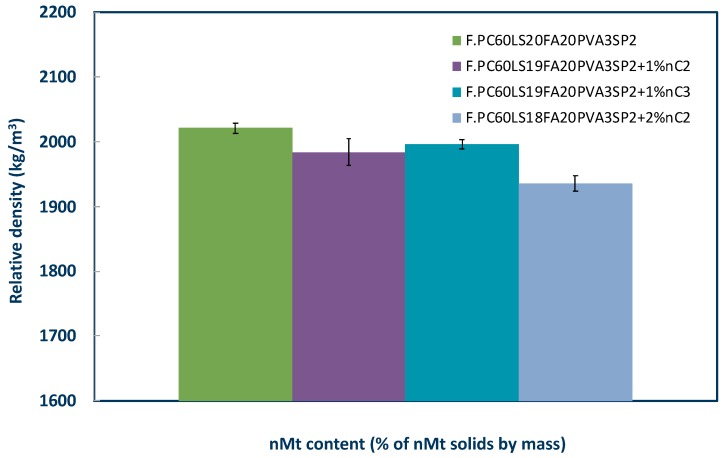
Effect of nMt type and concentration on long term relative density of nMt and fibre reinforced cementitious nanocomposites based on F.PC60LS20FA20PVA3SP2.

Relative density was measured after month 3 for the 1% nMt and fibre reinforced cementitious nanocomposites. As illustrated in Figure 14, relative density measurements exhibited low scattering, particularly nC3 and similar values of relative density for the 1% nMt content. It should be noted that as the nMt content increased, the relative density decreased. This can be explained by the clustering of nMt and fibre particles within the cement paste, which, in turn, is expected to increase the porosity, as also depicted by the BSE micrographs. 

### 3.5. Mercury Intrusion Porosimetry (MIP) of nMt and Fibre Reinforced Cementitious Nanocomposites Based on F.PC60LS20FA20PVA3SP2

In mercury intrusion porosimetry (MIP) the size of a pore system is inversely proportional to the pressure needed to force mercury into the pore system. Hence, MIP can provide information on porosity at the nanolevel and is particularly useful at comparative assessments of the pore refinement that may be taking place in the modified cement pastes [35]. The total number of pores, the median pore area diameter and the average pore diameter may be estimated, although studies have suggested that MIP pore size distribution estimates are actually unreliable [36]. Moreover, the samples need to be dried prior to the execution of the measurement and drying procedures generally influence the results and only relatively small samples can be analysed, therefore, not necessarily representative of the bulk. 

**Figure 15:**
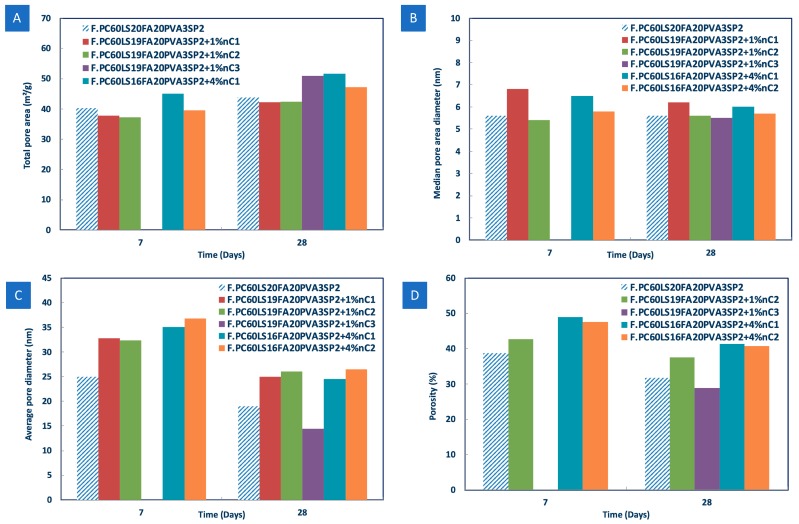
Effect of nMt type on (**A**) the total pore area, (**B**) the median pore area diameter, (**C**) the average pore diameter and (**D**) the porosity of fibre cements.

In fact, MIP measurements carried out for the 1% and 4% nC content, showed that the addition of high quantities of nMt yielded an increase in the total pore area (Figure 15A), the median pore area diameter (Figure 15B), the average pore diameter (Figure 15C), and porosity (Figure 15D) of fibre cement pastes. These results gave further justification to the hypothesis that nMt addition should be limited to approximately 1%. Furthermore, it is interesting to note that the 1% nC3 modified fibre cement paste was the only nMt modified sample that showed lower porosity than the reference paste at day 28. It is acknowledged, though, that later ages should be investigated with a number of techniques to ascertain this evidence. 

### 3.6. Water Permeability of nMt and Fibre Reinforced Cementitious Nanocomposites Based on F.PC60LS20FA20PVA3SP2

**Figure 16:**
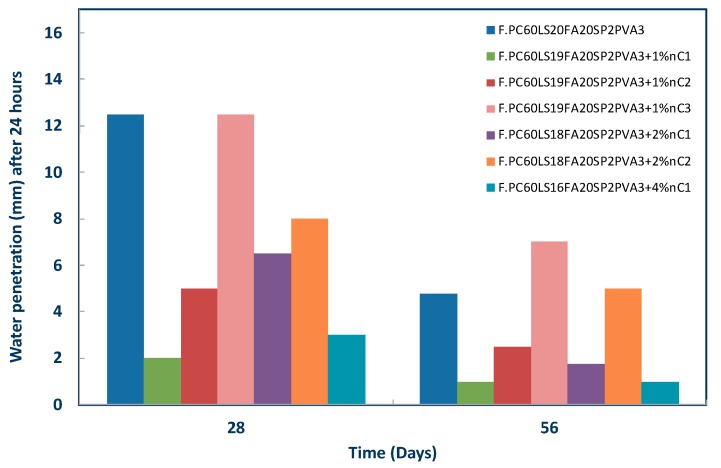
Effect of nMt type and concentration on the impermeability of nMt and fibre reinforced cementitious nanocomposites based on F.PC60LS20FA20PVA3SP2.

The nMt and fibre reinforced cementitious nanocomposites were also tested for impermeability. The theory suggesting that inorganic nMt adsorbs more water is verified (Figure 16), with the binders containing organomodified nMt’s exhibiting better performance than the reference paste at both early and later ages. Once again, the higher nMt concentrations are not effective, as was the case with the MIP analyses. These results are directly related to the flexural strength performance, as well, leading to the conclusion that higher nMt dosages, create clusters, increase porosity and in total, add localized weaknesses in the volume of cement pastes.

**Figure 17:**
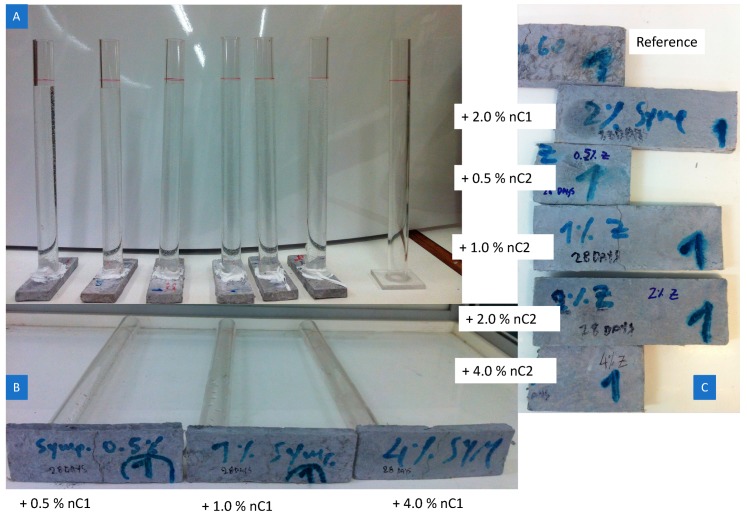
(**A**) Example of impermeability test, (**B**) difference in water absorption between the various concentrations of the nC1 and fibre reinforced cementitious nanocomposites and (**C**) difference in water absorption between the various concentrations of the nC2 and fibre reinforced cementitious nanocomposites based on F.PC60LS20FA20PVA3SP2.

After the test was completed, specimens were turned over to record the presence of moisture through the body of the specimen to the face of the bottom surface. Snapshots in Figure 17 depict the pronounced moisture as the amount of nC1 or nC2 increases and the overall restricted permeability of nC1.

## 4. Conclusions

All experiments presented in this paper were carried out under an FP7 (EU funded) project with the acronym FIBCEM. The full title of the project was “Nanotechnology-enhanced Extruded Fibre Reinforced For Cement-based Environmentally Friendly Sandwich Material for Building Applications”. Within FIBCEM, a significant number of research papers have been published by the authors on ternary, quaternary or quinary pastes, which did not include fibres or superplasticizers [4,5,8,24,32]. The current paper presents results on the effect of different nMt types on nMt-fibre reinforced binders with reduced PC content. The investigation concerned alterations of flexural strength, pozzolanic reactivity, density, water penetration and porosity at different concentrations of nMt. The following conclusions were deduced:

nC1 modified pastes showed significantly higher Ca(OH)_2_ consumption, but, the weakest performance in terms of strength. This can be attributed to the limited exfoliation of the OMMT. Interestingly though, the addition of nanosilica was shown to offer improvements even in this type of nMt. The optimal content was determined at 1% of solids by total mass of binder.

nC1 being the least advantageous nMt dispersion can be optimized by the addition of nanosilica particles. Therefore, according to the strength requirements, LnS can be added to the formulations presented in this paper for additional strength.

Although nC2 provided higher flexural strengths, compacting the nC2 and fibre reinforced binders was difficult due to reduced flowability. This, possibly lead to increased clustering of the various particles. This in turn, caused significant variations in strength. The optimal content was again determined at 1% of solids by total mass of binder.

The dispersion that provided the greatest pozzolanic reactivity, was nC3. The additional C–S–H provided by the pozzolanic reaction possibly lead to the improved flexural strength. In terms of water permeability, the nC3 reinforced binders were found to adsorb significant amounts of water compared to the reference binder.

Further research could shed more light on the microstructure of these binders and X-ray tomography (CT) scan can be suggested for more detailed characterization. The effect of nMt in such permeable fibre reinforced cementitious nanocomposites on freeze-thaw resistance should also be studied.

Mass production could be the next step in research, but can only be considered if compaction issues are minimized. For this, the fibre content is advised to be limited to 2%. 

Nano swelling may have taken place, filling the capillary pores [37]. Nanomontmorillonites’ LD C–S–H porosity could possibly be detectable by the MIP, [38], however further studies with different techniques should verify such hypothesis.

An additional advantage of nMt and fibre reinforced cementitious nanocomposites (as shown in the snapshots provided) is that their surface can be level with careful compaction, eliminating traffic induced surface ravelling.

The inorganic nMt dispersion, generally overlooked, can offer strength and porosity enhancement, while allowing water to penetrate, rendering it potentially suitable for the cementitious binder of pervious substrates. Although a number of standardized tests apply for the case of bituminous formulations suitable for pavements such as tests for the aggregates used or design methods such as the Marshall method whose principal purpose is to determine the most appropriate binder content for a given aggregate grading, there are no standardized methods for non-bituminous formulations. Therefore, the presented suite of experiment could serve as the basis for a broader research on such mixes enhanced with fibres and nMt’s.

Clearly, as the use of inorganic nMt for producing construction materials with specific properties is gaining momentum, more research is required for the verification of such applications. In conclusion, the results presented in this paper pushed knowledge ahead by offering the option of inorganic nMt, for permeable binders of reduced (beyond the permissible by the codes limits) PC content.

## Figures and Tables

**Table 1:** Nano-montmorillonite (nMt)-fibre reinforced quaternary formulations—Mix proportions % by total mass of solids.

Sample	PC (%)	LS (%)	FA (%)	nMt (%solids)	SP (%)	PVA (%)	W/S
F.PC60LS20FA20PVA3SP2	60	20	20	0.0	2	3	0.3
F.PC60LS19.5FA20PVA3SP2 + 0.5% nMt	60	19.5	20	0.5	2	3	0.3
F.PC60LS39PVA3SP2 + 1%n Mt	60	19	20	1.0	2	3	0.3
F.PC60LS18FA20PVA3SP2 + 2% nMt	60	18	20	2.0	2	3	0.3
F.PC60LS16FA20PVA3SP2 + 4% nMt	60	16	20	4.0	2	3	0.3

**Table 2:** nMt and LnS modified fibre reinforced quinary cement paste formulations - Mix proportions % by total mass of solids.

Sample	PC (%)	LS (%)	FA (%)	nC1 (%solids)	LnS (%solids)	SP (%)	PVA (%)	W/S
F.PC60LS20FA20PVA4SP2	60	20	20	0.0	0.0	2	4	0.3
F.PC60LS19.5FA20PVA4SP2 + 2.0% nC1	60	19.5	20	2.0	0.0	2	4	0.3
F.PC60LS17.5FA20PVA4SP2 + 2.0% nC1 + 0.5% LnS	60	17.5	20	2.0	0.5	2	4	0.3
F.PC60LS17FA20PVA4SP2 + 2% nC1 + 1.0% LnS	60	17	20	2.0	1.0	2	4	0.3
